# Evaluation of the Inferior Alveolar Canal Course in Relation to Fully and Partially Impacted Lower Third Molar Roots via Cone-Beam Computed Tomography

**DOI:** 10.7759/cureus.74021

**Published:** 2024-11-19

**Authors:** Anna Pogorzelska, Agnieszka Mielczarek, Piotr A Regulski, Mike Lee, Kazimierz Szopinski

**Affiliations:** 1 Department of Dental and Maxillofacial Radiology, Medical University of Warsaw, Warsaw, POL; 2 Department of Conservative Dentistry, Medical University of Warsaw, Warsaw, POL

**Keywords:** cone-beam computed tomography, inferior alveolar canal, mandibular canal, mandibular third molars, tooth impaction

## Abstract

Introduction: Impacted mandibular third molars are frequently encountered in dentistry. As extraction is often the treatment of choice, knowledge of the proximity and relationship of the inferior alveolar canal (IAC) with the tooth is essential. This study was designed to determine the course of the IAC in cases of impacted lower third molars.

Methods: Cone-beam computed tomography (CBCT) imaging of 103 patients (mean age: 37 years, range: 22-82 years) with 140 impacted lower third molars from 2016 to 2017 was evaluated. The number of roots, type of impaction according to Winter's classification, positional relationship of the IAC in relation to the roots, and shape of the canal were examined.

Results: The most common type of impaction was mesioangular, two-rooted molars. Most often, the canal ran below and in contact with the root, and the canal was most commonly oval-shaped. The type of impaction and the number of roots did not depend on sex. Single-rooted (p = 0.025) and distoangularly impacted (p = 0.019) molars were more common in older patients. There was a significant relationship between the number of roots and the canal running between the roots (p < 0.0001).

Conclusions: There was no relationship between the shape of the IAC and the number of roots or type of impaction. Based on these results, the interesting correlation between age, type of impaction, and number of roots requires further investigation and explanation. The frequency of canals in contact with the root further emphasizes the importance of CBCT imaging for preoperative visualization.

## Introduction

Tooth impaction occurs when a tooth fails to erupt in the dental arch within the expected timeframe. It can be caused by various factors, such as inadequate arch length, the presence of a cyst or tumour, and/or teeth crowding. In particular, impaction of the lower third molar is most commonly observed [1], leading to a number of complications, including pericoronitis, pain, tenderness, caries (due to unfavourable position and difficulties of hygiene maintenance), resorption of the adjacent teeth, and/or compression of adjacent structures [2]. When these teeth are extracted, the relationship between the impacted tooth and the inferior alveolar canal (IAC) undoubtedly plays a crucial role in treatment planning to minimize complications caused by iatrogenic damage to the inferior alveolar nerve (IAN), leading to temporary or permanent paresthesia.

Although panoramic imaging is routinely performed on patients, allowing an overview of the condition of the oral cavity that cannot be visualized clinically, it is not always sufficient for assessing impacted teeth. Most importantly, the two-dimensional image does not allow differentiation of buccolingual relationships. To counter these shortcomings, cone-beam computed tomography (CBCT) has gained popularity in recent years because of its ability to obtain images in axial, coronal, and sagittal views. While traditional computed tomography (CT) has the same ability, the use of CBCT is more prevalent in dentistry due to lower radiation and faster scanning despite poor imaging of soft tissues. CBCT employs a cone-shaped X-ray beam, rotating to various degrees around the patient according to the field of view selected, and divides the image into voxels [3]. In the context of impacted third molars, CBCT allows the determination of the pathway of the IAC in relation to the tooth root, measurement of the distance between the root and canal, and visualization of the relationship with adjacent structures, yielding valuable information for the dentist.

In this study, we aimed to assess the course of the IAC in relation to fully and partially impacted lower third molars. A further aim was to investigate whether the course of the IAC correlates with sex, age, type of impaction, number of roots, and shape of the canal.

## Materials and methods

This retrospective study was conducted at the Department of Dental and Maxillofacial Radiology, Medical University of Warsaw, Poland. Between January 2016 and December 2017, CBCT scans were obtained for 1,571 patients using the Scanora 3Dx CBCT system (Soredex, Tuusula, Finland). The voxel size and slice thickness were 0.2 mm for all scans. The field of view (FOV) depended on clinical indications; three sizes of FOV were used: 10x15 mm (imaging of the maxilla and mandible), 5×10 cm (imaging of the mandible), and 5×5 cm FOV (imaging of specific localized). The exposure parameters were consistent for all scans, set at 90 kVp and 10 mAs. Clinical indications for CBCT imaging included preoperative evaluation and detailed anatomical visualization.

All studies in the specified period were checked against the following inclusion criteria: (1) FOV, including the entire second and third mandibular molars and the IAC on one or both sides; (2) the presence of partially or completely impacted lower third molars; and (3) the development of at least three-quarters of the root length (stage R3/4 according to Moorees'). Sufficient imaging of the second molar region was required to assess the inclination of the impacted tooth using Winter's classification [4]. Exclusion criteria included the presence of focal lesions (such as periapical cysts or tumours), root development not reaching three-quarters, and lack of visibility of the crown, root, and IAC.

After screening all the CBCT studies against the inclusion criteria, a total of 103 patient CBCT studies met the requirements and were therefore included in this study. Thus, the study comprised 140 impacted mandibular third molars (39 males and 61 females; mean age 36.36 years, range 22-82 years).

The scans meeting the criteria were viewed in the computer software CS 3D Imaging v. 3.10.4 (Carestream Dental, Atlanta, USA) by two independent examiners (one dental radiologist with 10 years of experience and one radiologist with 30 years of experience), where the images were analyzed in the sagittal, coronal, and axial cross sections.

**Figure 1:**
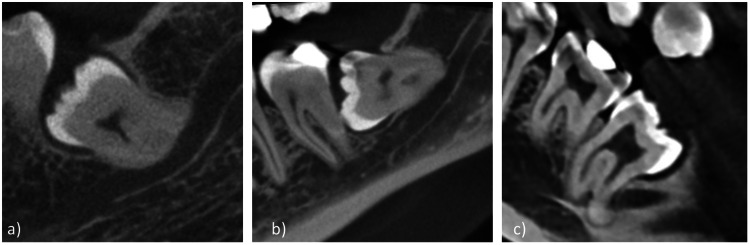
Images of various CBCT scans showing impacted third molars classified as (a) mesioangular, (b) horizontal, or (c) distoangular CBCT: cone-beam computed tomography

The number of roots and the pattern of impaction were first determined. The pattern of the impacted tooth was assessed using Winter's classification (mesioangular, distoangular, horizontal, or vertical) [4], which is based on the angle of the impacted tooth to the long axis of the second molar (Figure 1).

**Figure 2:**
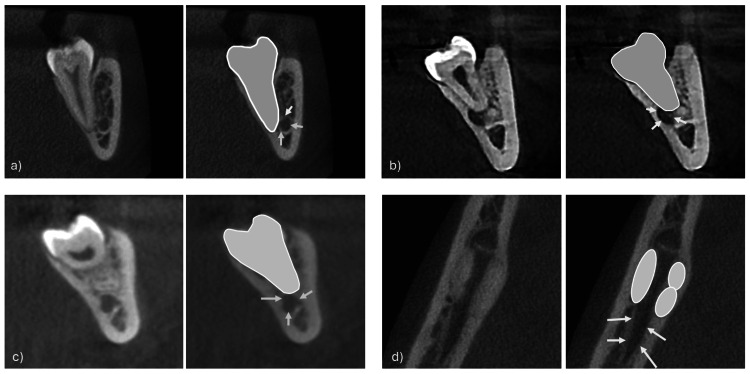
Assessing the inferior alveolar canal in relation to the roots. The inferior alveolar canal may be classified as buccally positioned, lingually positioned, inferiorly positioned, and/or positioned between the roots (a) Buccal, (b) lingual and inferior, (c) inferior, or (d) between roots

**Figure 3:**
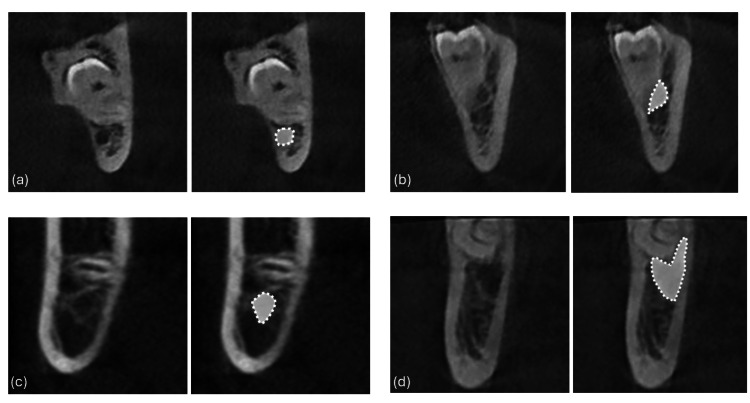
The cross-sectional shape of the inferior alveolar canal was assessed The shapes are classified as (a) circular, (b) oval, (c) teardrop, or (d) dumbbell

Subsequently, the positional relationship between the IAN and the root was analyzed. The IAC may be classified as one or more of the following: buccally positioned, lingually positioned, inferiorly positioned, or positioned between the roots (Figure 2). Finally, the shape of the IAC was classified into one of four types: round, oval, dumbbell, or teardrop (Figure 3).

χ2 tests and ANOVA were used to explore the correlation between the course of the IAC and various factors. A significance threshold of p < 0.05 was established for our analyses.

## Results

The data were gathered from 103 CBCT images of different individuals and included 140 lower third molars. Table 1 lists the basic patient demographics. Among the 103 patients, the mean age was 34.4 years, with more females (61/103; 59.2%) represented in the sample. The left (67/140; 47.86%) and right (73/140; 52.14%) third molars were almost equally represented in the sample.

**Table 1:** Demographics and basic information

Mean age (standard deviation)	34.4 ± 7.3	
Gender (n = 103)	Male	42
	Female	61
Side of impacted mandibular third molar (n = 140)	Left	67
	Right	73

Table 2 shows the number of roots in molars and statistics based on age and sex. The most commonly observed teeth were two-rooted molars (95/140; 67.86%), followed by one- and three-rooted teeth. There were no teeth with more than three roots. There was a statistically significant relationship with age, with single-rooted teeth being more common in older patients and three-rooted teeth being more common in younger patients (p = 0.019), while no statistically significant relationship was found with sex (p = 0.31).

**Table 2:** Number of roots in relation to sex and age Statistically significant for p < 0.05

Number of Roots	Total N (%)	By Gender N (%)	χ^2^ Statistic	p-value (Gender)	Age (Mean ± SD, Years)	F-statistic	p-value (Age)
1	29 (20.71%)	Male: 12 (41.4%), female: 17 (58.6%)	2.31	0.31	41.48 ± 13.11	4.08	0.019
2	95 (67.86%)	Male: 43 (45.3%), female: 52 (54.7%)	-	-	35.43 ± 11.45	-	-
3	16 (11.43%)	Male: 4 (25%), female: 12 (75%)	-	-	32.63 ± 7.25	-	-
Total	140 (100%)	Male: 42 (30%), female: 61 (70%)	-	-	36.36 ± 11.69	-	-

Table 3 shows the distribution of the type of impaction in relation to age and gender. Mesioangularly positioned teeth were the most common (58/140; 41.43%), followed by vertically (41/140; 29.29%), horizontally (34/140; 24.29%), and distoangular (7/140; 5%) impacted teeth. The order of frequency according to the type of impaction did not differ based on sex (mesioangular most common, distoangular least common), and there was no significant relationship between sex and the type of impaction (p = 0.75). Distoangular impaction was observed in older patients (average 46.29 years), and mesioangular impaction was observed in younger patients (average 34.38 years). Similar to the findings regarding the number of roots, there was a statistically significant relationship between age and the type of impaction (p = 0.025).

**Table 3:** Type of impaction in relation to gender and age Statistically significant for p < 0.05

Type of Impaction	Total N (%)	By Gender N (%)	χ^2^ Statistic	p-value (Gender)	Age (Mean ± SD, Years)	F-statistic	p-value (Age)
Mesioangular	58 (41.43%)	Male: 26 (44.8%), Female: 32 (55.2%)	1.19	0.75	34.38 ± 10.04	5.22	0.025
Distoangular	7 (5.00%)	Male: 4 (57.1%), Female: 3 (42.9%)	-	-	46.29 ± 17.18	-	-
Horizontal	34 (24.28%)	Male: 13 (38.2%), Female: 21 (61.8%)	-	-	39.18 ± 11.97	-	-
Vertical	41 (29.29%)	Male: 16 (39.0%), Female: 25 (61.0%)	-	-	35.15 ± 11.73	-	-
Total	140 (100%)	Male: 39 (27.9%), Female: 61 (72.1%)	-	-	36.36 ± 11.69	-	-

Table 4 summarizes the course of the IAC. Most often, the canal ran inferiorly to the root. Lingually positioned canals were more common than buccally positioned canals. The least common course of the canal was running inter-radicularly. A statistically significant relationship exists between canals running inter-radicularly and three-rooted teeth, although this seems evident due to the anatomy.

**Table 4:** Course of the canal in relation to the number of roots and type of impaction Statistically significant for p < 0.05

Canal Course	Total N (%)	1-rooted N (%)	2-rooted N (%)	3-rooted N (%)	χ^2^ Statistic (Roots)	p-value (Roots)	Mesioangular N (%)	Distoangular N (%)	Horizontal N (%)	Vertical N (%)	χ^2^ Statistic (Impaction)	p-value (Impaction)
Buccal	38 (19.0%)	7 (18.4%)	26 (68.4%)	5 (13.2%)	29.62	<0.0001	22 (57.9%)	1 (2.6%)	5 (13.2%)	10 (26.3%)	47.85	<0.0001
Lingual	50 (25.0%)	11 (22.0%)	36 (72.0%)	3 (6.0%)	-	-	1 (2.0%)	4 (8.0%)	0 (0.0%)	5 (10.0%)	-	-
Inter-radicular	21 (10.5%)	1 (4.8%)	9 (42.9%)	11 (52.4%)	-	-	5 (23.8%)	7 (33.3%)	2 (9.5%)	33 (33.3%)	-	-
Inferior	108 (54.5%)	25 (23.1%)	71 (65.7%)	12 (11.1%)	-	-	10 (9.3%)	18 (16.7%)	8 (7.4%)	27 (25.0%)	-	-
Total	217 (100%)	44	142	31	-	-	38	30	15	75	-	-

Tables 5, 6 delineate the contact of the canal. The canal was in contact with the root in 88.57% of the patients (124 impacted molars out of 140). Canals running an inferior course were statistically associated with canals without contact. There was no significant relationship between the number of roots and the type of impaction.

**Table 5:** Contact with the canal in relation to the number of roots and type of impaction Statistically significant for p < 0.05

Contact	Total N (%)	1-rooted N (%)	2-rooted N (%)	3-rooted N (%)	χ^2^ Statistic (Roots)	p-value (Roots)	Mesioangular N (%)	Distoangular N (%)	Horizontal N (%)	Vertical N (%)	χ^2^ Statistic (Impaction)	p-value (Impaction)
With	124 (88.57%)	28 (22.6%)	80 (64.5%)	16 (12.9%)	5.67	0.06	55 (44.4%)	5 (4.0%)	28 (22.6%)	36 (29.0%)	5.60	0.13
Without	16 (11.43%)	1 (6.2%)	15 (93.8%)	0 (0.0%)	-	-	3 (18.8%)	2 (12.5%)	6 (37.5%)	5 (31.2%)	-	-
Total	140 (100%)	29	95	16	-	-	58	7	34	41	-	-

**Table 6:** Contact with the canal in relation to the course of the canal Statistically significant for p < 0.05

Contact	Buccal N (%)	Lingual N (%)	In Between Roots N (%)	Inferior N (%)	χ^2^ Statistic	p-value
With	37 (29.8%)	49 (39.5%)	21 (16.9%)	92 (74.2%)	12.14	0.007
Without	1 (6.2%)	1 (6.2%)	0 (0.0%)	16 (87.5%)	-	-

Tables 7, 8 show the relationships between the shape of the canal and the number and type of root impacted. When examining the shape of the IAC on CBCT scans, we found that oval-shaped canals were the most dominant, constituting more than half the sample (81/140; 57.86%). Circular canals were the second most common (21/140; 15.00%), although the number of circular canals did not differ much from that of dumbbell-shaped (19/140; 13.57%) or teardrop-shaped canals (19/140; 13.57%). We could not find a statistically significant relationship between the shape of the canal and the number of roots or type of impaction.

**Table 7:** Shape of the canal in relation to the number of roots Statistically significant for p < 0.05

Number of Roots	Oval N (%)	Dumbbell N (%)	Teardrop N (%)	Circle N (%)	Total N (%)	χ^2^ Statistic	p-value
1	20 (24.7%)	1 (5.3%)	5 (26.3%)	3 (14.3%)	29 (20.7%)	6.59	0.36
2	54 (66.7%)	14 (73.7%)	11 (57.9%)	16 (76.2%)	95 (67.9%)	-	-
3	7 (8.6%)	4 (21.1%)	3 (15.8%)	2 (9.5%)	16 (11.4%)	-	-
Total	81 (57.9%)	19 (13.6%)	19 (13.6%)	21 (15.0%)	140 (100%)	-	-

**Table 8:** Shape of the canal in relation to the type of impaction Statistically significant for p < 0.05

Type of Impaction	Oval N (%)	Dumbbell N (%)	Teardrop N (%)	Circle N (%)	Total N (%)	χ^2^ Statistic	p-value
Mesioangular	27 (33.3%)	12 (63.2%)	8 (42.1%)	11 (52.4%)	58 (41.4%)	15.00	0.09
Distoangular	6 (7.4%)	0 (0.0%)	1 (5.3%)	0 (0.0%)	7 (5.0%)	-	-
Horizontal	24 (29.6%)	5 (26.3%)	4 (21.1%)	1 (4.8%)	34 (24.3%)	-	-
Vertical	24 (29.6%)	2 (10.5%)	6 (31.6%)	9 (42.9%)	41 (29.3%)	-	-
Total	81 (57.9%)	19 (13.6%)	19 (13.6%)	21 (15.0%)	140 (100%)	-	-

## Discussion

The lower third molars in this study were most commonly mesioangularly impacted, followed by vertically impacted, which is similar to the findings of related studies [4-11]. A CBCT study revealed that horizontally impacted teeth were most prevalent in Saudi [12] and Japanese [13] populations and that vertically impacted teeth were most prevalent in the Chinese [14] population. Several panoramic studies [1,15,16] have reported vertically impacted teeth as the most common, followed by mesioangularly impacted teeth. However, other panoramic studies agreed with our findings [17-19]. The percentage of distoangular impacted teeth (4.93%) in our study was less prevalent than that in similar studies [4,11,15-17]. Gümrükçü et al. [15] reported that 25% of the impacted mandibular third molars were distoangular, and Patel et al. [7] reported 13%. While differences in the prevalence and type of impaction can be attributed to demographic differences, a study conducted in a similar geographic area (Szczecin, Poland) [11] revealed that 39.04% of mandibular third molars were impacted distoangularly.

The greater percentage of females in our sample is consistent with studies examining impacted mandibular third molars [5,7,9,10,15,20]. Several propositions have been suggested about this observation, ranging from women being more attentive to their bodies and seeking medical care [5] to differences in jaw growth between genders [7]. However, it should be noted that there were also studies reporting a male preponderance [12,18]. In our study, there were no significant differences regarding the course of the mandibular canal, type of impaction, or number of roots according to sex. Gender variation regarding the pattern of impaction has been investigated in several studies, which have shown variable findings. The findings of Obuekwe and Enabulele [20] on Nigerian patients were consistent with our findings, while the study by Al-Anqudi et al. [21] conducted in Oman concluded that mesioangular impaction was significantly more likely in males and distoangular impaction was more likely among females.

The alignment of two-rooted mandibular third molars was most commonly reported in other studies [6]. The number of three-rooted teeth reported was significantly greater than that reported in studies on lower third molars conducted in Korea [22] and India [23], although the samples in those studies were not limited to impacted teeth. Our study found a significant correlation between canals running inter-radicularly and three-rooted molars, suggesting that anatomical relationships influence canal courses in these cases. The statistically significant correlation between age, the type of impaction, and the number of roots in our study is worth noting. With an average age of 34.4 years, distoangularly impacted teeth (average 46.3 years) and one-rooted teeth (average 41.5 years) tended to be found in older patients.

Ryalat et al. [24] reported a greater incidence of vertically impacted lower third molars (70-100 degrees) in the older age group and a significantly lower incidence of horizontally impacted molars in the older age group than in the younger age group. An increased retromolar area was also observed in older patients. Studies have reported a positive correlation between age and mandibular canal-to-root distance for third molars [25]. Although none of the studies directly replicated our findings, this suggests that the stomatognathic system is constantly growing and modifying, providing insight into how the impaction angle can differ over time. However, this finding does not explain the differences in the number of roots according to age. Age is correlated with an increased risk of postoperative complications, including neurosensory disturbances after tooth removal, which may be attributed to changes in bone physiology and hypercementosis leading to a challenging removal [2,26].

Numerous studies have explored risk factors for nerve injury following third molar extraction. Radiographic signs on a pantomogram, such as loss of cortication, darkening of the root(s), and interruption of the radiopaque canal outline, are reported as risk factors [18,27]. Other studies have examined demographic factors such as gender [28] and age [26,29] as risk factors for temporary and permanent paresthesia, while several studies have not identified a greater risk associated with the female gender [25]. A lingually located IAC on CBCT is reportedly a risk factor for neurosensory disturbances after extraction [19,30] and is associated with an increased incidence of root contact [31]. Close root contact is considered a high-risk factor for IAN damage after extraction [27]. Currently, common radiographic classifications for assessing tooth impaction of third molars, including Winter's and Pell & Gregory's classification, do not take into account the IAN, although a few have been proposed [32,33] that account for the buccolingual position and presence of root-canal contact. Furthermore, cross-sectional canal shape is often investigated in studies via CBCT, as it is suggested in the literature that the risk of IAC injury in the literature is closely associated with canal shape [34], a feature that can only be evaluated via three-dimensional imaging.

We found that the IAN was most commonly positioned inferior to the roots and was more often lingual than buccal relative to the roots. This finding aligns with other CBCT studies examining the IAC relationship [5,33,35,36]. The majority (124 out of 140) of the impacted mandibular third molars were in contact with the IAC, similar to other reports [5,17,31]. Nevertheless, the potential for selection bias must be acknowledged, as CBCT imaging is often indicated only when an intimate relationship between the canal and tooth is found in panoramic imaging. Other literature studies reported a lower contact rate [27,33]. A lack of contact between the canal and roots was strongly associated with an inferior position of the canal in our study, while others have reported a relationship between canal contact and a lingually positioned nerve [5,12]. We were not able to establish a statistical relationship with Winter's classification, similar to other studies [5].

In line with other studies, oval and circular cross-sectioned canals constituted the majority of our sample, in line with other studies. In most studies [10,30], there were more teardrop-shaped canals than dumbbell-shaped canals, while in our sample, they were of equal numbers. An oval/circular cortical outline of the mandibular canal was associated with lower rates of contact between the IAN and the roots of the third molar. Dumbbell-shaped canals were found to have an increased risk of IAN injury after extraction [19,30], as well as teardrop-shaped canals [37]. We were not able to correlate the canal shape with the number of roots or the type of impaction.

As CBCT imaging becomes more accessible and popular in dentistry, numerous studies have explored its necessity and effectiveness. Currently, recommendations from the European Academy of Dentomaxillofacial Radiology (EADMFR) do not support routine preoperative imaging of the third molar, recommending CBCT imaging only for specific clinical queries that cannot be addressed with conventional (panoramic, periapical) imaging [36]. A meta-analysis by Telles-Araújo et al. [38] and independent studies [36,39] indicated that additional CBCT exams do not reduce the risk of IAN injury or neurosensory disturbances, leading instead to comparable long-term patient outcomes. However, studies comparing the effectiveness of panoramic imaging and CBCT often highlight differences in tooth evaluation leading to changes in treatment decisions, including impaction type according to Winter, Pell & Gregory [40], and the number of roots [41]. In addition, prognostic factors such as location, shape, and absence of cortication were deemed more reliable than signs used in panoramic imaging [19]. It should be noted that CBCT examination serves a broader purpose than observing root-canal relations, and visualization of the surrounding anatomy greatly aids in the dentist's comprehension and confidence in the case. In our study, a lack of correlation was evident between Winter's classification and the position of the canal, necessitating the use of CBCT scans to determine the relationship.

Further work is needed to correlate the findings with clinical implications, such as a change in the technique of tooth extraction when there is an intimate tooth-canal relationship. A decreased incidence of neurosensory disturbances has been reported with coronectomy [42], orthodontic extrusion, and piezosurgery [43]. While anatomy and radiographic findings are risk factors for IAN injury, the technique and expertise of the operator can also play a role. In addition, no clinical outcomes were examined (e.g., whether the patient had complications after extraction) in this study. While root-canal contact suggests a greater risk for IAN injury, this does not mean that it will occur; vice versa, nerve injury may occur despite a lack of contact. The sample size was also a weakness of this study.

This study presents several limitations. The relatively small sample size and the restriction to a specific geographic and demographic group may limit the generalizability of the findings. Furthermore, the lack of longitudinal data on post-extraction outcomes limits our ability to determine which anatomical characteristics might have the greatest impact on postoperative complications. Future research incorporating larger, more diverse samples and prospective follow-up would be valuable to confirm these findings and enhance their clinical applicability.

## Conclusions

In conclusion, the use of CBCT for impacted lower third molars provides essential insights into root-canal relationships, significantly aiding surgical planning and reducing the risk of complications. This study found that 88.57% of impacted molars were in direct contact with the IAC, with mesioangular impaction being the most common type. These findings reaffirm the importance of CBCT imaging, particularly when panoramic imaging indicates a close relationship between the canal and the tooth.

## References

[1] Hupp JR, Tucker MR, Ellis E (2018). Contemporary Oral and Maxillofacial Surgery. https://shop.elsevier.com/books/contemporary-oral-and-maxillofacial-surgery/hupp/978-0-323-55221-9.

[2] Rizqiawan A, Lesmaya YD, Rasyida AZ, Amir MS, Ono S, Kamadjaja DB (2022). Postoperative complications of impacted mandibular third molar extraction related to patient's age and surgical difficulty level: a cross-sectional retrospective study. Int J Dent.

[3] Whaites E (2002). Essentials of Dental Radiography and Radiology. https://shop.elsevier.com/books/essentials-of-dental-radiography-and-radiology/whaites/978-0-7020-7688-6.

[4] Santos KK, Lages FS, Maciel CA, Glória JC, Douglas-de-Oliveira DW (2022). Prevalence of mandibular third molars according to the Pell & Gregory and Winter classifications. J Maxillofac Oral Surg.

[5] Quirino de Almeida Barros R, Bezerra de Melo N, de Macedo Bernardino Í, Arêa Leão Lopes Araújo Arruda MJ, Meira Bento P (2018). Association between impacted third molars and position of the mandibular canal: a morphological analysis using cone-beam computed tomography. Br J Oral Maxillofac Surg.

[6] Khojastepour L, Khaghaninejad MS, Hasanshahi R, Forghani M, Ahrari F (2019). Does the Winter or Pell and Gregory classification system indicate the apical position of impacted mandibular third molars?. J Oral Maxillofac Surg.

[7] Patel S, Mansuri S, Shaikh F, Shah T (2017). Impacted mandibular third molars: a retrospective study of 1198 cases to assess indications for surgical removal, and correlation with age, sex and type of impaction-a single institutional experience. J Maxillofac Oral Surg.

[8] Mohanty R, Rout P, Singh V (2020). Preoperative anatomic evaluation of the relationship between inferior alveolar nerve canal and impacted mandibular third molar in a population of Bhubaneswar, Odisha, using CBCT: a hospital-based study. J Maxillofac Oral Surg.

[9] Alkhader M, Jarab F (2016). Visibility of the mandibular canal on cross-sectional CBCT images at impacted mandibular third molar sites. Biotechnol Biotechnol Equip.

[10] Tassoker M (2019). Diversion of the mandibular canal: is it the best predictor of inferior alveolar nerve damage during mandibular third molar surgery on panoramic radiographs?. Imaging Sci Dent.

[11] Jaroń A, Trybek G (2021). The pattern of mandibular third molar impaction and assessment of surgery difficulty: a retrospective study of radiographs in East Baltic population. Int J Environ Res Public Health.

[12] Shujaat S, Abouelkheir HM, Al-Khalifa KS, Al-Jandan B, Marei HF (2014). Pre-operative assessment of relationship between inferior dental nerve canal and mandibular impacted third molar in Saudi population. Saudi Dent J.

[13] Momin MA, Matsumoto K, Ejima K (2013). Correlation of mandibular impacted tooth and bone morphology determined by cone beam computed topography on a premise of third molar operation. Surg Radiol Anat.

[14] Chen Y, Liu J, Pei J, Liu Y, Pan J (2018). The risk factors that can increase possibility of mandibular canal wall damage in adult: a cone-beam computed tomography (CBCT) study in a Chinese population. Med Sci Monit.

[15] Gümrükçü Z, Balaban E, Karabağ M (2021). Is there a relationship between third-molar impaction types and the dimensional/angular measurement values of posterior mandible according to Pell & Gregory/Winter classification?. Oral Radiol.

[16] Yilmaz S, Adisen MZ, Misirlioglu M, Yorubulut S (2016). Assessment of third molar impaction pattern and associated clinical symptoms in a central Anatolian Turkish population. Med Princ Pract.

[17] Khouri C, Aoun G, Khouri C, Saade M, Salameh Z, Berberi A (2022). Evaluation of third molar impaction distribution and patterns in a sample of Lebanese population. J Maxillofac Oral Surg.

[18] Deshpande P, V Guledgud M, Patil K (2013). Proximity of impacted mandibular third molars to the inferior alveolar canal and its radiographic predictors: a panoramic radiographic study. J Maxillofac Oral Surg.

[19] Shiratori K, Nakamori K, Ueda M, Sonoda T, Dehari H (2013). Assessment of the shape of the inferior alveolar canal as a marker for increased risk of injury to the inferior alveolar nerve at third molar surgery: a prospective study. J Oral Maxillofac Surg.

[20] Obuekwe O, Enabulele J (2017). Gender variation in pattern of mandibular third molar impaction. J Dent Oral Disord Ther.

[21] Al-Anqudi SM, Al-Sudairy S, Al-Hosni A, Al-Maniri A (2014). Prevalence and pattern of third molar impaction: a retrospective study of radiographs in Oman. Sultan Qaboos Univ Med J.

[22] Park JB, Kim N, Park S, Ko Y (2013). Evaluation of number of roots and root anatomy of permanent mandibular third molars in a Korean population, using cone-beam computed tomography. Eur J Dent.

[23] Somasundaram P, Rawtiya M, Wadhwani S, Uthappa R, Shivagange V, Khan S (2017). Retrospective study of root canal configurations of mandibular third molars using CBCT- part-II. J Clin Diagn Res.

[24] Ryalat S, AlRyalat SA, Kassob Z, Hassona Y, Al-Shayyab MH, Sawair F (2018). Impaction of lower third molars and their association with age: radiological perspectives. BMC Oral Health.

[25] Puciło M, Puciło A, Safranow K, Nowicka A (2021). The influence of age, sex, and tooth type on the anatomical relationship between tooth roots and the mandibular canal. Imaging Sci Dent.

[26] Leung YY, Cheung LK (2011). Risk factors of neurosensory deficits in lower third molar surgery: an literature review of prospective studies. Int J Oral Maxillofac Surg.

[27] Akter M, Rahman QB, Uddin MW, Kundu GC, Banik S, Imon AA (2016). Pre-operative assessment of impacted mandibular third molar and inferior alveolar canal using orthopantomography and cone beam computed tomography. Bangabandhu Sheikh Mujib Med Univ J.

[28] Miloro M, DaBell J (2005). Radiographic proximity of the mandibular third molar to the inferior alveolar canal. Oral Surg Oral Med Oral Pathol Oral Radiol Endod.

[29] Sklavos A, Delpachitra S, Jaunay T, Kumar R, Chandu A (2021). Degree of compression of the inferior alveolar canal on cone-beam computed tomography and outcomes of postoperative nerve injury in mandibular third molar surgery. J Oral Maxillofac Surg.

[30] Tachinami H, Tomihara K, Fujiwara K, Nakamori K, Noguchi M (2017). Combined preoperative measurement of three inferior alveolar canal factors using computed tomography predicts the risk of inferior alveolar nerve injury during lower third molar extraction. Int J Oral Maxillofac Surg.

[31] Chaudhary B, Joshi U, Dahal S, Sagtani A, Khanal P, Bhattarai N (2020). Anatomical position of lower third molar in relation to mandibular canal on cone-beam computed tomography images in a tertiary care hospital: a descriptive cross-sectional study. JNMA J Nepal Med Assoc.

[32] Wang WQ, Chen MY, Huang HL, Fuh LJ, Tsai MT, Hsu JT (2015). New quantitative classification of the anatomical relationship between impacted third molars and the inferior alveolar nerve. BMC Med Imaging.

[33] Maglione M, Costantinides F, Bazzocchi G (2015). Classification of impacted mandibular third molars on cone-beam CT images. J Clin Exp Dent.

[34] Ueda M, Nakamori K, Shiratori K (2012). Clinical significance of computed tomographic assessment and anatomic features of the inferior alveolar canal as risk factors for injury of the inferior alveolar nerve at third molar surgery. J Oral Maxillofac Surg.

[35] Liu ZL, Jiang ES, Cui LY, Li JX (2023). Cone-beam computed tomography analysis on the relationship between the mandibular third molar and the position of the mandibular canal in Koreans from the Yanbian area and the Han people. Int J Clin Pract.

[36] Matzen LH, Berkhout E (2019). Cone beam CT imaging of the mandibular third molar: a position paper prepared by the European Academy of DentoMaxilloFacial Radiology (EADMFR). Dentomaxillofac Radiol.

[37] Wang D, Lin T, Wang Y, Sun C, Yang L, Jiang H, Cheng J (2018). Radiographic features of anatomic relationship between impacted third molar and inferior alveolar canal on coronal CBCT images: risk factors for nerve injury after tooth extraction. Arch Med Sci.

[38] de Toledo Telles-Araújo G, Peralta-Mamani M, Caminha RD, de Fatima Moraes-da-Silva A, Rubira CM, Honório HM, Rubira-Bullen IR (2020). CBCT does not reduce neurosensory disturbances after third molar removal compared to panoramic radiography: a systematic review and meta-analysis. Clin Oral Investig.

[39] Korkmaz YT, Kayıpmaz S, Senel FC, Atasoy KT, Gumrukcu Z (2017). Does additional cone beam computed tomography decrease the risk of inferior alveolar nerve injury in high-risk cases undergoing third molar surgery? Does CBCT decrease the risk of IAN injury?. Int J Oral Maxillofac Surg.

[40] Mendonça LM, Gaêta-Araujo H, Cruvinel PB (2021). Can diagnostic changes caused by cone beam computed tomography alter the clinical decision in impacted lower third molar treatment plan?. Dentomaxillofac Radiol.

[41] Peker I, Sarikir C, Alkurt MT, Zor ZF (2014). Panoramic radiography and cone-beam computed tomography findings in preoperative examination of impacted mandibular third molars. BMC Oral Health.

[42] Monaco G, D'Ambrosio M, De Santis G, Vignudelli E, Gatto MR, Corinaldesi G (2019). Coronectomy: a surgical option for impacted third molars in close proximity to the inferior alveolar nerve-a 5-year follow-up study. J Oral Maxillofac Surg.

[43] Wang Y, Xu Y, Chi Z, Mao R (2022). Compressing inferior alveolar nerve impacted mandibular third molar: a comparison of three techniques. Oral Surgery.

